# Collectanea: Miscellaneous

**Published:** 1834-10-01

**Authors:** 


### MISCELLANEOUS.

### PHYSIOLOGY OF THE FŒTUS.

It is of the utmost importance to bear in mind the great distinction which exists between the independence of the fœtus, *quoad* life, and its dependence, *quoad* nutrition, in respect to the mother. The former state is secured by a total separation of the two circulations (maternal and fœtal); the latter by the close reciprocal contact of the organs of those circulations. Thence it is that we find the fœtus to live on, notwithstanding that its connexion with the mother has been partially, and sometimes even wholly severed; while, on the other hand, we cannot help admitting that, albeit this independence, the influence of the mother over the fabric of her offspring is unquestionable.

Here are two important positions. I have mentioned my experiments on the intact ova of the genus cat, to illustrate the first of them; and Dr. Prevost has since come to my assistance, with as strong a case in further support of it. (*Mémoires de la Societé de Physique de Genève*, t. iv. part 1.) This gentleman having observed an ovum still alive in the uterus of a ewe, which was a short time advanced in gestation, removed it, and placed it upon a warm glass plate exposed to the rays of the sun, and attentively examined it with the microscope. The beatings of the heart became more lively. He noticed the blood arise to the surface of the chorion from the fœtus, there ramify plentifully, and, by anastomosing vessels, return to two of the larger trunks, which were the veins of the embryo. He concluded, therefore, that the ovum was an isolated substance.

In proof of the accuracy of the second position we have equally strong evidence, founded on experiments. Magendie introduced camphor into the veins of a pregnant bitch, and he found that the blood of the fœtus had, at the expiration of a quarter of an hour, acquired distinctly the smell of that drug. (*Physiology*, 2d edit., 1825.) Quadrupeds carrying young were made to take with their food four ounces of madder-root. The colouring matter of that substance was found to have passed from the mother to the fœtus; as all the serum of the blood of the latter, the urine, the liquor amnii, the teeth, and the bones, were tinged with it. (*Dr. Mussy*, 1829.*

In 1827 I undertook, at the request of Sir E. Home, a set of experiments on the human subject, with a view to ascertain the truth of my second position. Six gravid patients of one of the lying-in institutions under my direction, who required the constant use of aperient medicines, were instructed, towards the close of their time of gestation, to take at night, for a period which averaged about a week, from ten to fifteen grains of rhubarb in powder.

* American Journal of the Medical Sciences, November, 1829.

After two or three days the effect was visible in the colour and smell of the urine of the patients previous to their confinement, and, in one of them, in the colour and smell of the transpiration also. During the labour, care was taken to preserve part of the amnionic fluid in a cup, the umbilical vessels were suffered to bleed on the side of the child after their recision, and the blood set apart so as to separate the serum, which was obtained in small quantity. Lastly, the first urine of the child was collected in sufficient portions. Each of these secretions appeared distinctly tinged by the yellow root, and bore the smell of it. When carbonate of magnesia was mixed with the fluids, their colour became lateritious, and a reddish sediment was thrown down, evincing the presence of the drug which the mother had ingested. (*MS. Notes.*) —*Dr. Granville's Graphic Illustrations of Abortion and the Diseases of Menstruation.*

### QUAIN'S ANATOMICAL PLATES.

We fear that it will be long before this useful work is finished, for the last Number on our table (XV.) is dated July 1. The period of five years, two months, and a fortnight, which we formerly assigned to the completion of the work, must therefore be still further extended. Our younger readers, (for it is to them alone that we venture to address advice of this kind,) when they shall have risen to the rank of professors and authors, will, we trust, abstain from this modern error of publishing books in infinitesimal fractions; for it is a system pregnant with disappointment and mortification to author, bookseller, and reader. When a sufficient portion of the plates shall be before us, we shall not fail to give a faithful account of them.

### A STURDY HYPOCHONDRIAC.

I finish with a short story, which may seem ridiculous, but it is true, and shows the whimsicalness (if I may use the expression,) of the disease. A certain fellow of a college, by too much indulging a sedentary life, was so severely afflicted with this disorder, that he was at length obliged to take to his bed; and his hypochondriacism gradually rose to that pitch, that he declared himself at the point of death. In that fit he ordered his passing knell to be rung in a church not far from his chambers; which was accordingly done, but in so bungling a manner, in his opinion, (for he had been a famous ringer in his younger days,) that, in a violent passion, he jumped out of bed, ran to the church, chid the sexton, and told him he would show him the true way of ringing. Whereupon he grasped the rope, and fell to work with such vehemence, that he soon wrought himself into a muck sweat; then returned to bed, in order to die contented. But he was disappointed, for the exercise restored him to life and health. Thus, as Hippocrates formerly observed, contraries are remedies of contraries.—*Mead, of the Hypochondriacal Disease.* (Works, p. 557. Ed. 1762.)

### INFLUENCE OF THE MOON.

A more uncommon effect of this attractive power is related by the learned Kirckringius. He knew a young gentlewoman whose beauty depended upon the lunar force; insomuch that at full moon she was plump and handsome, but in the decrease of the planet so wan and ill-favoured, that she was ashamed to go abroad, till the return of the new moon gradually gave fulness to her face, and attraction to her charms. (*Observ. Anatomic.* 92.) If this seems strange, it is indeed no more than an influence of the same kind with that which the moon has always been observed to have upon shellfish, and some other living creatures; for, as the old Latin poet Lucilius says,

> Luna alit ostrea, et implet echinos, muribu' fibras

> Et pecui addit. (*Apud A. Gellium,* lib. xx. c. 8.)

And after him Manilius:

> Si submersa fretis, concharum et carcere clausa,
> Ad lunæ motum variant animalia corpus.
> (*Astronomia,* lib. ii. ver. 93.)

*Mead; Influence of Sun and Moon upon Human Bodies.* (Works, p. 186. Ed. 1762.)

### GINSENG.

The Ginseng, *Panex quinquefolium,* has from immemorial ages been extolled in China as a universal medicine, or panacea, whence its present generic name, which is a compound of παν ακος, and signifies a remedy for all things. But, notwithstanding its foreign name, it is very little used in Europe; perhaps, its high-sounding title, having led to undue expectations, may have caused it to fall into unmerited neglect; for it does seem, when fresh, to be an agreeable stimulant and tonic. Père Jartroux says, that the most celebrated physicians of China have written volumes on the *Gen-seng,* which they affirm to be able to ward off or to remove fatigue, to invigorate the enfeebled frame, to restore the exhausted animal powers, to make old people young, and, in a word, to render man immortal; (this saving clause being however added,) "if anything on earth can do so." Hence the name *Gen-seng, Jin-chen,* or *Nind-sin,* which signifies "wonder of the world," or the "dose for immortality." Osbeck says, the Chinese take it every night and morning in their tea or soup, and that he never looked into the apothecaries' shops but they were always selling ginseng. The plant is a native of North America, as well as of Chinese Tartary: it grows chiefly in desert places difficult of access, or, at least, the venders tell strange tales of dangers encountered by those who collect the root, perhaps with the design of enhancing its value; and it has been known to cost its weight in gold.—*Burnett's Outlines of Botany.*

MUMMIES.

The body of the mummy being washed, according to Herodotus, after its immersion in the solution of natron, and thus all superfluous salt likely to attract moisture removed, the bandages are to be applied. All mummies, however, were not bandaged. Many had only the covering of a mat, which surrounded them. Denon states that he saw bodies without bandages in the Necropolis of Thebes, and Belzoni saw the bodies of two females lying on the ground in a corner of a chamber in one of the tombs in the valley of Biban el Molonk without any bandages; they were well preserved, their hair long and flowing in tresses. The quantity of bandages on some mummies have been computed to consist of not less than one thousand ells. Abd'Allatif says that in some mummies more than one thousand yards have been used, and that the Bedouins were in the habit of taking it away to make vestments, or to sell for the manufacture of paper for the grocers. The bandages on Mr. Davidson's mummy weighed twenty-nine pounds and a half. The mummy of Horseisi thirty-five pounds and a half. Herodotus states that it was profane for the Egyptians either to be buried in woollen garments, or to use them in their temples. "Les Prêtres ne pouvoient porter ni des habits de laine, ni des souliers de cuir; parceque la laine engendre la vermine, et que le cuir vient d'une bête morte."

The bandages generally employed in the enveloping of the mummies have been supposed to be of cotton. Jomard says that in the catacombs at Philæ linen was clearly to be detected; it was of a coarse description, and he conceives answered for the poorer class of people. Rouyer also noticed a difference in the texture of the bandages. Jomard has gone more particularly into the manufacture of it. * * *

Dr. Ure has been so good as to make known to me that which I conceive to be the most satisfactory test of the absolute nature of flax and cotton, and in the course of his microscopic researches on the structure of textile fibres he has succeeded in determining their distinctive characters. From a most precise and accurate examination of these substances he has been able to draw the following statement: "The filaments of flax have a glassy lustre when viewed by daylight in a good microscope, and a cylindrical form, which is very rarely flattened. Their diameter is about the two thousandth part of an inch. They break transversely with a smooth surface, like a tube of glass cut with a file. A line of light distinguishes their axis, with a deep shading on one side only, or on both sides, according to the direction in which the incident rays fall on the filaments.

"The filaments of cotton are almost never true cylinders, but are more or less flattened and tortuous; so that when viewed under the microscope they appear in one part like a riband from the one thousandth to the twelve hundreth part of an inch broad, and in another like a sharp edge or narrow line. They have a pearly translucency in the middle space, with a dark narrow border at each side, like a hem. When broken across, the fracture is fibrous or pointed. Mummy-cloth tried by these criteria in the microscope appear to be composed both in its warp and woof-yarns of flax, and not of cotton. A great variety of the swathing fillets have been examined with an excellent achromatic microscope, and they have all evinced the absence of cotton filaments."—*Pettigrew's History of Egyptian Mummies.*

### MANUFACTURE OF MUMMIES.

Frequent deceptions have been practised in the manufacture of mummies, as we have seen in a previous chapter. I purchased three mummies at the sale of the museum of the late Mr. Heaviside, to whom they had been presented as Egyptian. I have opened two of them, one apparently that of an infant of very tender age, the other that of a child about six or seven years. The former consisted literally of saw-dust, bundles of rags of various descriptions, and a portion of stick to serve the place of the spine. There were some of the vertebræ of a cat mixed up with the dust. The bandages enclosing this rubbish were of the true Egyptian character, and had, doubtless, been taken off from other and real mummies in the country. The face was formed of linen covered with plaster of Paris, and carefully made out. The second specimen was of a similar manufacture, the face of the same description, but the contents of the head consisted of the bones of a human skull about the age of eight years, which led me in the first instance to think that it had been a genuine mummy; but, upon examination, I found three temporal bones, which was conclusive on the matter. Around the body of this figure were the proper kind of bandages, and some linen with hieroglyphics painted upon it. The contents consisted of bones of various kinds, some belonging to a human fœtus, others of a more advanced age, and some bones of the monkey, and the entire hand of a small species of this animal. M. Jomard mentions the making up of mummies similar to these.—*Ibid.*

### AN AUTOPSY BY STEAM.

One of the steam-carriages on the New Road, we observe, has been styled "The Autopsy;" an ominous name, some medical men may think, having notions of death and dissection in their head the moment they hear of it; and some may even fancy it absurd. But we do not agree with them; for autopsy is surely just as good a name for a steam-coach as for the thing it has latterly been applied to in medicine. It properly means what one has seen with one's own eyes, just as autograph does what one has written with one's own hand: and how is it, in any one respect, more applicable to a post-mortem than to a steam-coach? Whenever, in future, we see autopsy prefixed to the report of a post-mortem examination, we shall scarcely be able to divest our minds of the steam-coach, with its hurry, noise, and smoke. We wish the worthy steam proprietors would borrow a few more hard and unmeaning words from us; for, thanks to the medico-pedantic spirit prevailing in some quarters, we have latterly had a heavy overflow of them.—*Med. Gazette.*

### COTTON MILLS.

I wish to give my evidence; I wish to say, that as I use up in my concern one nineteenth part of the cotton consumed in Great Britain, that I think it is right that I should declare my opinion about a proposed alteration in the law.

First of all, with regard to the beating of children in mills, my rules are these, and I am very strict about them: it is forbidden; but children must be corrected to a certain extent; we will not turn floggers, and when children require severe correction we turn them away, first speaking to the parents several times, in order that they may correct them if they think proper; we allow no beating with straps or ropes, and if a child is beaten, we have the child, the spinner, and overlooker up face to face; but the only trouble that I have about beating in my mills is with the spinners; I cannot always prevent them from doing it to the extent that I wish, for children require correction every now and then, and the difficulty is to keep it from being excessive. If the child has been improperly dealt with, I propose to the spinner either to pay a fine for the benefit of the child, or I threaten to send for a warrant, and have him up before a magistrate. I have had three cases of this kind in about six months. I have a great number of hands in my employ, about 1,500; I cannot and do not know them all. It never can be the interest of a master that the children should be beaten. The other day there were three children ran away; the mother of one of them brought him back again and asked us to beat him; that I would not permit; she asked us to take him again; at last I consented, and then she beat him; he was a card-room child.

Now, as regards temperature, I really do not know what the temperature of my mills is. It is our own interest to prevent the mills from being too hot, because it costs us money to heat them. The spinners may open the windows when they like in the spinning-rooms, and shut them when they like; the temperature need not be more than moderate; in the card-rooms we send in no steam at all.

Now my opinion is, that whatever bill is passed, it should be a tight one, and such as can never be evaded; it should be equal and efficient; and for this it must be a restriction on the moving power; then if that is not granted, we ought to have a public officer, a warden, and he must enforce the law at his discretion, so as to place all on the same footing as to the hours they work. I think that water-mills should not have any privileges of working up time beyond those which steam mills have; and I would restrict every mill whatever to this, that it should never work more than twelve hours a day; in other words, that there should be no fetching-up of lost time at all, except on the day on which the loss is made.

Regarding the character of females engaged in cotton-mills. My father was a medical man, and he made me study medicine for three years, and I am fond of the subject of medicine. I cannot say conscientiously that I think that the females are at all worse in their conduct than those engaged in other occupations, save and except, that I think that where a great number of them get together in cotton mills they are not so good as when they are more separated; if there is any difference, it proceeds rather from the business requiring them to be congregated, than from laxity of discipline or other incentives to bad conduct, or anything in the trade itself; the children and the adults are blanched by the confinement in-doors in artificial temperature, but their health is quite as good as the average in other trades.—*Factories' Enquiry. Evidence of John Bolling, esq., in Part I. Supplementary Report.*

[This overgrown spinner's calculating humanity, his hypothesis that "it never can be the interest of a master that the children should be beaten," will remind our readers of the West India planters' cant, who used to protest that it never could be their interest that the negroes should be cruelly treated; yet, when it came to counting, and their paternal chastisements were arithmetically estimated, 150,000 lashes were found to be laid on, in one year, in one colony (Demerara). In the next page, we find Jonathan Ambray, operative spinner, giving an account of the cruelties to which he was subjected at various mills; and, after detailing the shameful mnnner in which he was treated at Mr. Peter Marsland's, in Stockport, he adds, "Mr. Marsland knew nothing of all this, but he pressed for a quantity of work, which could not be got without hands using some sort of severity." (P. 175.) To be sure; the mill-owner, like the slave-holder, does not say, kick me those children, flog me those blacks, but contents himself with demanding an almost impossible quantity of yarn, (as the kindred spirit does* of sugar,) which comes to the same thing. *Qui facit per alium, facit per se*, says common sense.

We hope, too, that there are but few practitioners of physic so fascinated by manufacturers' smiles, or so demoralized by manufacturers' gold, as to pretend to believe that persons blanched by the confinement in-doors in artificial temperature are in good health.—Ed. *Med. Quart. Rev.*]

THE STUDIES OF A PHYSICIAN.

His first care will be to make himself fully acquainted with the curious structure of the human frame; the functions of every part in a state of health, and its deviations from that sane and healthy condition under diseases; the symptoms of which he must next learn to discriminate with the nicest care. After this, he will inform himself profoundly of the various remedies of our art, whether they be supplied by the botanist or the chemist, or come from whatever other source; and, lastly, with the appropriate application of medicine to particular disease. I forbear to enter more minutely into

* This was written before the 1st of August. We trust we may now say *did*, instead of *does*.

the order in which lectures should be attended. Every medical school has its own arrangements.

But it may not be unnecessary to guard the student against being seduced to pay a disproportionate attention to any one branch of the course. To become exclusively the botanist or chemist, or even the anatomist, where the one great object is the cure of diseases, will narrow both his resources and his mind, and will make him incur the risk of failure in the end. Philosophy, to an intellect now so well prepared to investigate its hidden truths, and to make discoveries in the ample field of general science, presents, it must be admitted, most seductive charms. But the example of Hercules, in the interesting story of his choice, must govern the student's conduct; and he will do well to remember the rebuke of Menedemus in the play, "Chreme! tantumne in re tuâ otii datur, aliena ut cures, eaque ad te quæ nihil attinent?"

No: the cure of diseases, I repeat it, is the physician's object, and he must not allow anything to divert his eye from that great mark. Botany and chemistry, enchanting as they are, only furnish tools to the hand of the workman. They are but subsidiary instruments, wherewith to execute, not to form great designs.

Nor is it safe to attach himself to the consideration of some one particular disease. If exclusive and particular attention be given to one malady, with the ambition of acquiring early fame by it, suspicion will arise that this physician's mind is less comprehensive than is necessary to take in all the objects within the horizon of science. Nor is it less impolitic and prejudicial in another point of view; for if any one case turn out ill in the hands of such a person, his good name will be put into jeopardy immediately, on the conclusion (lame and impotent it may be,) that if he could not cure a disease to which he had paid such extraordinary attention, how should he master another which had not duly engaged his mind?

Nor must he addict himself to any particular system, nor swear by the opinions of any master. He must exercise his own judgment, and be ready to profit of occasions, "scire uti foro," according to the Roman proverb; and to accommodate himself to circumstances as they arise, either by the adoption of a new treatment by new remedies, or by the use of accredited ones in new and unusual doses, remembering another remark of that great master of human nature, Terence: "Nunquam ita quicquam bene subductâ ratione ad vitam fuit, quin res, ætas, usus semper aliquid apportent novi, aliquid moneant, ut illa quæ scire te credas, ea quæ putaris prima, in exercendo ut repudias." (Vide *Adelphi.*)—*Sir Henry Halford on the Education and Conduct of a Physician.*

### OCULISTS IN THE SEVENTEENTH CENTURY.

It is gratifying to find that the same Richard Banister, of whom I have been speaking, and who wrote really a very good book on Diseases of the Eye,—it is, I say, pleasing to find such an individual zealous in the exposure of all unprofessional practices, and apparently as tenacious of his professional dignity and privileges as a M.R.C.S. of the present day. He has devoted a long section of his book to the exposure of what he terms "proud quacksalving mountebankes, that would undertake all cures, and performe few;" and intimates that "such are they that promise to make blind people see, deafe people heare, and to cure the stone and rupture by cutting;" and thus expresses his virtuous indignation against this cunning fraternity of knaves: "In the methodicall practice and cure of blind people, by couching of cataracts, our English oculists haue always had an especiall care, according to arts, to couch them within doores, out of the open aire, to preuent further danger. Yet some of these mountebanks take their patients into open markets, and there, for vaine-glories sake, make them see, hurting the patient, only to make the people wonder at their rare skill. Some others make scaffolds, on purpose to execute their skill vpon, as the Frenchmen, and the Irishman did in the Strand, making a trumpet to be blowne before they went about their work. But these were not long suffered to vse these lewd courses, before they were called before the company of chirurgiens: being sharply reprooued, soone left the city, and their abusiue practice."—*Middlemore's Introductory Lecture on Diseases of the Eye.*

### THE PULSE IN HORSES.

The state of the pulse is highly important in almost all diseases. Naturally, it beats about forty times in a minute. A few beats either below or above this standard need not be noticed, as it will vary even in health in different horses; but when it comes to ascend to fifty, and to mount beyond this, it furnishes reason to suspect that the operations of the body are in some way or other disturbed. As regards its frequency, and in reference to the natural standard of forty, the pulse may be slow, or it may be quick. I have found the pulse myself as low as twenty-four: I have heard Mr. Sewell say, he has met with it not more than fourteen.—*Percivall's Hippopathology.*

### TEMPERAMENT.

In the writings of Galen there is a treatise expressly composed to prove that the characters of men depend upon their temperaments. But it is in the works of modern writers that we find this doctrine most fully developed, and made a foundation for a division of human characters. According to Hoffmann, the choleric temperament, by peculiarity of organization, disposes men to precipitate and impetuous conduct, to anger, audacity, impatience, temerity, quarrels, sedition, and the like. On the other hand, the slow progress of blood through the vessels of the meninges, which is the result of its crassitude in melancholics, renders such persons timid, slow in business, anxious, suspicious, with difficulty of forming or uttering opinions. The sanguine, by a happier temperament, are rendered cheerful and free from care. A too abundant serosity causes the phlegmatic to be lazy, somnolent, and torpid. Certain temperaments qualify men for particular situations in life. Melancholic men, says Hoffmann, should be the king's ministers and counsellors; choleric persons should be appointed generals, foreign ambassadors, orators, conductors of all business requiring energy and despatch. Sanguine men are fit for courtiers; but individuals who have the misfortune to be of the phlegmatic temperament, being quite incompetent to any elevated condition, must be made common soldiers or labourers, and condemned to the lowest employments.* The same writer applies the doctrine of temperaments to nations, and explains by it their peculiarities. It is wonderful how ready a belief was given to notions so ill founded, and to what an extent they were carried. The very learned Abbate Hervas finds a sufficient reason in the difference of temperaments for the conquests which northern nations have so often made in southern countries.† The English, however, says this sensible but occasionally quaint writer, though belonging to the stock of colder climates, have become "*dal troppo bevere liquori gagliardi*,"—in temperature unlike the other inhabitants of the north.

It is extremely improbable that an opinion should have held its ground for so many ages among men of observation, especially on a subject requiring no abstruse research, without some foundation at least in fact. The doctrine of temperaments is true to a certain extent, and has ever been confirmed by an appeal to experience. In order to be convinced of this, let any person compare individuals who display the characters of the sanguine temperament in a high degree, with others who have strongly marked signs of the melancholic. There is no doubt that among the first will be found many in proportion who have a lively and cheerful temper, great sensibility, excitable if not strong passions; and, among the latter, persons who are, if not sullen and dejected, (for such qualities are morbid extremes,) yet calm and disposed to seriousness and reflection, rather than to mirth and gaiety; at the same time tenacious of impressions once excited in their minds, and capable of fixed and steady attention. These characteristic differences may be referred to greater or less degrees of sensibility, or to a more or less excitable condition of the nervous system, depending perhaps, in the first place, on the circulation of blood, the apparatus for which is (as we learn from the greater vigour with which the function is performed,) more fully developed in the sanguine than in the melancholic. No facts are more familiar in physiology than the inti-

* Hoffmann, de Temperamento Fundamento Morborum, § 10.

† The work of Hervas, in nineteen quarto volumes, contains an epitome of human knowledge. It is entitled "Idea del universo, che contiene la Storia della Vita del Uomo, Elementi cosmografici, Viaggio estatico al Mondo planetario, e Storia della Terra." (Cesena, 1780.) Natural philosophy, physiology, anatomy, and other physical sciences; history, politics, statistics, are treated of in turn, with a prodigious extent of information. The last volumes of the work consist of treatises on philology, and contain much original information on the history of languages not elsewhere to be found.

mate connexion between organic sensibility and a free circulation, or than the increase or diminution of feeling which results from warmth and increased vascular action, and from coldness and torpor, and the retirement of blood from the surface of the body and the organs of sense. States of the mind are so connected with affections of the body, that it is impossible for any person who considers these and the many other analogous facts which present themselves, to doubt that with each temperament particular mental qualities must be associated, although it is manifest that many writers have indulged their fancy on this subject, and have gone into more full and minute details than experience will establish.—*Dr. Prichard, in the Cyclopædia of Medicine, Part XXI.*

### SARRACENNIA.

The several species of Sarracennia already known, which do not exceed six, with two or three varieties, are all natives of the bogs and swamps of North America. Of their properties there is nothing decidedly ascertained, and they are chiefly interesting from bearing the curious pitcher-like organs already described, which contain water, to which, in dry weather, it is affirmed, birds and other animals resort to assuage their thirst. The lids are said to close over the mouths of the urns in dry weather, to prevent the evaporation of the water, which is probably designed to furnish the plant with supplies when the marshes are exhausted. These pitchers also contain large numbers of dead flies and other insects, which, when putrefying, give out an intolerable odor, that renders the plants offensive; but the decomposition of the animal matter affords a supply of rich and very nutritious food, probably essential to the well-being of the Sarracenniæ, as they are furnished with such curious organs to entrap and retain it; organs which have been supposed to be amongst the earliest adumbrations of a stomach.—*Burnett's Outlines of Botany.*

### DEFECTS IN MEDICAL EDUCATION.

1st. The *competency of the teachers* is subjected to no test or proof; and the establishment of schools, and the appointment of teachers, are left to the caprice or predilections of the treasurers and governors of hospitals, or are the results of private speculation and individual self-interest.

2dly. Relatively to the *general practitioner*, the great majority of the profession, the utterly *inadequate period* assigned for the acquisition of the competent skill and knowledge, when in two years the students are expected to obtain all the requisites for safe practisers of a most complex and difficult art.

3dly. The *negligence* too often permitted in enforcing even a decorous, much less a close and unremitting attendance during their studies.

4thly. The *insufficiency of the examinations* of which a diploma or licence is to be the reward, as a test of the competency of the candidate; this too being the only pledge to the public that the candidate for practice is duly qualified.

No objection will, we apprehend, be offered to this view, from a consideration of the education appropriate to each class or branch of the profession, since the negligences and defects are in their consequences common to all. It applies especially to the most numerous class, the general practitioners, in respect of whom we might to the above list of defects add the injurious enforcing of apprenticeships, that most grievous obstacle to a liberal and truly professional education; the removal of which, together with the final separation of the medical practitioner, of whatever degree or title, from the trader in drugs and servile compounder of recipes and prescriptions, is to be earnestly wished, alike for the patient's sake and the practitioner's,—by all who do not advocate the retention of names under a total change and revolution of the persons and circumstances. — *Green's Suggestions respecting Medical Reform.*

### MEDICAL RESPONSIBILITY IN FRANCE.

The Visigoths had a law, that if a physician were called in to a case, and took charge of it, he was bound to effect a cure. If the patient died, the physician was immediately delivered up to the friends of the deceased, that they might do what they liked with him. Some of the French journalists complain piteously that the laws affecting the profession in France, at the present day, are not less severe. A case lately occurred at Evreux, in which a M. Thouret-Noroy, in consequence of alleged malapraxis, by accidentally injuring the brachial artery, so as to render amputation necessary, was mulcted in heavy damages; and, having appealed to a higher tribunal, had four hundred francs more laid on him, by way of interest on his former fine.—*Medical Gazette.*

### A CASE OF RABIES IN THE HORSE.

By Mr. C. Marshall, v.s., London.

On Thursday evening, the 17th of April, 1834, a message was sent to me late in the evening by Mr. Reynolds (the owner of the animal), that the old horse was very ill, and had something sticking in his throat. I was from home, and could not attend until Friday morning about seven o'clock. I found the horse foaming, breathing very laboriously, his tail erect, screaming dreadfully at short intervals, striking the ground with his fore feet, and perspiring most profusely. He would get into the manger, and strike his head against the wall, cringing and drawing himself up as though there was some obstruction in the œsophagus. He was continually biting the top of the stall, and when I approached him he tried to run at me. I considered him to be rabid, and advised Mr. Reynolds to have him destroyed. The pistols were got ready for his destruction; but, before we could use them, he broke the top of the manger, and came out of the stall with it hanging to the halter. He made immediately towards us, but, as we succeeded in getting out of his way, he turned into the next stall, and died instantly.

I inquired if they had any idea of his having been bitten by a dog, but they had not; I also inquired if they had noticed any thing unusual about the horse previously to the attack. They informed me that his spirits had been better than usual, that he appeared stronger, and that a lameness under which he laboured had left him for three or four days. He was at work within four hours of his seizure.

I examined him immediately after death, and found the upper and back part of his tongue, epiglottis, and the membrane lining the windpipe, in a high state of inflammation; the lungs also much gorged with blood. The brain, stomach, and every part of the viscera, were in perfect health.—*The Veterinarian, June.*

[Mr. W. Youatt afterwards gives a case in a spaniel, with the following post-mortem appearances: "Fauces much inflamed, extending over the membrane covering the dorsum of the epiglottis. Stomach very much inflamed, and throughout. Much dark-coloured slimy fluid in the stomach." We think the spinal chord ought to have been examined in both cases.—Ed. *Med. Quart. Rev.*]

### THE MANDRAKE.

*Atropa Mandragora.* Mandrake.—*Fl. Græc.* vol. iii. tab. 232. Μανδραγόρας.—*Diosc.* lib. iv. cap. 76, and lib. vi. cap. 16; also of *Theoph.* lib. ix. cap. 10. Μανδραγουρα hodie, et γοργογάνι quandoque apud Atticos. *Sibth.* Dioscorides relates that it was also called Κιρκαία, *Circæa*, because its root was used as a philter or love potion, ἐπειδὴ δοκει ἡ ῥιζα φίλτρων ἔιναι ποιητική. And Theophrastus says, that the root was of use, προς ὕπνον καὶ φίλτρα. For the same purpose mandrakes are mentioned in the book of *Genesis*, chap. xxx. v. 14—16. This plant even now possesses somewhat of its most ancient right in modern Greece:—Radicis frustula in sacculis gesta, pro *amuleto*, *amatorio* hodiè, apud juvenes Atticos in usû sunt. (*Fl. Græc.* p. 27.) And Maundrell states (p. 61), that the "women of Samaria are wont to apply it at this day, out of an opinion of its prolific virtue." The mandrake was properly called by Pythagoras ἀνθρωπόμορφος, from its root resembling the form of a man. See the figure in the *Flora Græca.* —*Hooker's Journal of Botany.*

### BURIAL IN HONEY.

Josephus records that the Jewish king Aristobulus, whom Pompey's partisans destroyed by poison, lay buried in honey till Antony sent him to the royal cemetery in Judæa.* The Assyrians placed the bodies of their dead in honey to preserve them from corruption. The Romans also used honey for the same purpose.† Abd'Allatif relates‡ an anecdote of a man who had found a sealed cruise, and, having opened it, he discovered it to contain honey, which he began to eat, until one of his companions observed a hair upon his finger, when the vessel was more closely examined, and a little child, all perfect, was withdrawn from it. The body was well preserved, and furnished with rich jewels and ornaments.

* Antiquitat. lib. xiv. c. 7.

† Montfaucon Antiquité Expliquée, tom. v. part ii., pp. 185, 186. ‡ p. 199.

